# Autoimmune Hepatitis in Brazilian Children: IgE and Genetic Polymorphisms in Associated Genes

**DOI:** 10.1155/2015/679813

**Published:** 2015-11-26

**Authors:** Léa Campos de Oliveira, Anna Carla Goldberg, Maria Lucia Carnevale Marin, Karina Rosa Schneidwind, Amanda Farage Frade, Jorge Kalil, Irene Kasue Miura, Renata Pereira Sustovich Pugliese, Vera Lucia Baggio Danesi, Gilda Porta

**Affiliations:** ^1^Laboratório de Medicina Laboratorial (LIM03), Hospital das Clínicas, Faculdade de Medicina, Universidade de São Paulo, 05403-000 São Paulo, SP, Brazil; ^2^Hospital Israelita Albert Einstein, 05652-900 São Paulo, SP, Brazil; ^3^Instituto de Investigação em Imunologia, Instituto Nacional de Ciência e Tecnologia, 05403-000 São Paulo, SP, Brazil; ^4^Laboratório de Imunologia, Instituto do Coração (InCor), Hospital das Clínicas, Faculdade de Medicina, Universidade de São Paulo, 05403-000 São Paulo, SP, Brazil; ^5^Laboratório de Histocompatibilidade e Imunidade Celular (LIM19), Hospital das Clínicas, Faculdade de Medicina, Universidade de São Paulo, 05403-000 São Paulo, SP, Brazil; ^6^Departamento de Hepatologia, Instituto da Criança, Hospital das Clínicas, Faculdade de Medicina, Universidade de São Paulo, 05403-000 São Paulo, SP, Brazil; ^7^Divisão de Alergia e Imunologia Clínica, Faculdade de Medicina, Universidade de São Paulo, 05403-000 São Paulo, SP, Brazil

## Abstract

Pediatric autoimmune hepatitis (AIH) patients present hypergammaglobulinemia, periportal CD8^+^ cytotoxic T cell infiltration, and cirrhosis. Autoantibody profile defines AIH types 1 and 2 in addition to strong association with HLA-DRB1. We previously detected increased IgE serum levels and sought to compare clinical and histological features according to IgE levels in AIH (*n* = 74, ages 1–14 years) patients. Additionally, we typed 117 patients and 227 controls for functional polymorphisms of IL4, IL13, IL5, and IL4RA genes involved in IgE switching and eosinophil maturation that might contribute to overall genetic susceptibility to AIH. Serum IgE levels were high in 55% of AIH-1, but only in 12% of AIH-2 (*P* = 0.003) patients. Liver IgE was present in 91.3% of AIH-1 patients. The A alleles at both IL13 rs20541 and IL4RA rs1805011 were associated with AIH-1 (*P* = 0.024, OR = 1.55 and *P* < 0.0001, OR = 2.15, resp.). Furthermore, individuals presenting homozygosis for the A allele at IL4RA rs1805011 and HLA-DRB1^*∗*^03 and/or ^*∗*^13 allele had sixfold greater risk to develop the disease (OR = 14.00, *P* < 0.001). The novel association suggests an additional role for IgE-linked immune response genes in the pathogenesis of AIH.

## 1. Introduction

Autoimmune hepatitis (AIH) is a chronic inflammatory disease characterized by progressive destruction of the hepatic parenchyma [1]. The disease displays female predominance and is considered rare in childhood, although it may occur in very young children [2]. The hallmark of the disease is the presence of circulating autoantibodies, defining two major subtypes: type 1 (AIH-1) [3, 4] and type 2 (AIH-2) [5]. Equally striking is the strong genetic susceptibility identified by specific MHC class II molecules, especially HLA-DRB1, which discriminates between the two types of AIH. Brazilian AIH-1 patients carry HLA-DRB1^*∗*^13 and/or HLA-DRB1^*∗*^03 whereas AIH-2 patients present mainly carry HLA-DRB1^*∗*^07 [6].

Hypergammaglobulinemia is a diagnostic feature of AIH but other immunoglobulins may be altered as well. Low IgA levels are particularly common in AIH-2 [7] and we have observed high IgE levels in children with AIH-1 [8]. Elevated serum IgE levels have been previously described in acute and chronic liver diseases usually linked to alcohol abuse or viral infection [9]. This phenomenon is traditionally linked to allergy, asthma, and atopy, but elevated IgE serum levels in specific autoimmune diseases have been increasingly acknowledged. To date, elevated IgE serum levels have been identified in Churg–Strauss vasculitis [10], sclerosing cholangitis [11], bullous pemphigus [12], autoimmune pancreatitis [13], and Grave's disease [14]. IgE seems also to play a role in the pathogenesis of rheumatoid arthritis contributing to the immune response against citrullinated proteins [15]. Atta et al. [16] also observed specific IgE antinuclear antibodies in systemic lupus erythematosus suggesting there is an important contribution to the pathogenesis of the disease. B lymphocyte switching to IgE is induced by IL4 and its neighbor gene IL13 [17], which form, together with IL5, a well-studied cytokine gene cluster (5q31.1) controlling TH2 type immune responses. IL4 is a pleiotropic cytokine essential for IgE synthesis by B cells and for T cell differentiation into a TH2 phenotype and upregulation of MHC class II expression. The functions of IL13 in immune surveillance and in TH2 type immune responses partially overlap with those of IL4. In addition to the classic TH2 pathway shared with IL4, IL13 has other important functions. IL13, together with IL5 [18], is a potent mediator of tissue fibrosis and tissue remodeling, as shown in experimental models of schistosomiasis [19]. A steadily increasing literature indicates that there is an important role for IL13 in the development of hepatic fibrosis, signaling through the IL13 receptor to induce collagen production by local fibroblasts [20] AIH-1 pediatric patients typically exhibit liver fibrosis, including most patients in our study. About 25% of AIH patients, despite treatment with corticosteroids, present progressive fibrosis, highlighting the importance of any gene which might be involved in this process [21]. In addition, both IL4 and IL13 genes harbor functionally relevant polymorphisms [22, 23].

Histological findings in AIH include typical piecemeal necrosis with infiltrating T lymphocytes. T cell-mediated cytotoxicity is believed to be the central mechanism responsible for hepatic damage, but other cells are involved. Typically, CD4^+^ helper T and B cells gather around portal tracts, whereas CD8^+^ cytotoxic T cells have a periportal distribution [24]. In addition to the abundant infiltrating mononuclear cells, plasma cells and eosinophils may also be present [1]. Interestingly, a previous study has highlighted the increased production of IL4 messenger RNA in AIH-1 liver biopsies in parallel with the expected increase in inflammatory interferon gamma and other proinflammatory cytokines [25]. These findings led us to try to identify additional factors involved in the autoimmune processes present in this liver disease, which might act either as prognostic disease markers or as novel targets for a therapeutic approach. To this end, we analyzed the major clinical manifestations and biopsies from Brazilian children grouped according to the AIH type and serum IgE levels. We also investigated, in the predominant AIH-1 group of patients, functional polymorphisms of the IL4, IL13, IL5, and IL4RA (IL4 receptor alpha chain) genes involved in IgE switching and eosinophil differentiation and maturation that we believe might contribute to overall genetic susceptibility to AIH.

## 2. Patients and Methods

A total of 141 patients diagnosed as AIH, according to the International Autoimmune Hepatitis Group Report [26], were studied. Patients were followed at the Pediatric Hepatology Unit of the Children's Institute, General Hospital, Faculty of Medicine, University of São Paulo in São Paulo, Brazil. Clinical, biochemical, and histological features of 74 AIH patients (61 with AIH-1 and 24 with AIH-2) aged 1 to 14 years were evaluated.

To increase statistical power for analysis of gene polymorphisms, we included a further 43 children with AIH-1 (a total of 117). Non-HLA matched siblings of bone marrow recipients from the same hospital and with similar social and ethnic background, without any autoimmune and/or other severe disease, were enrolled as healthy controls (HC, *n* = 227). Written informed consents were obtained from all participants and/or legal guardians, and the Internal Review Board of the University of São Paulo approved the study.

Laboratory liver tests, including alanine aminotransferase (ALT), aspartate aminotransferase (AST), alkaline phosphatase, gamma glutamyl transpeptidase (*γ*GT), albumin, *γ*-globulins, prothrombin, and total bilirubin, and autoantibody profiles were performed in all patients. Fecal samples collected in all patients were negative for parasitic infection. Radioallergosorbent test (RAST) for specific allergen against house dust, animal fur, food, and fungi was assayed by radioimmunoassay using Unicap100E (Pharmacia & Upjohn Company LLC, MI, USA) system. I. Immunoglobulins M, G, A, and E were assayed by nephelometry using a DADE Behring System Nephelometer BN 100 (Dade Behring Diagnostics Inc., Somerville, NJ). Serological tests for hepatitis A, B, and C were negative in all patients. Clinicians involved in this study ruled out other hepatic diseases such as alpha-1 antitrypsin deficiency and Wilson's disease.

Histological features of liver biopsies were graded semiquantitatively using the Brazilian Consensus for Histopathology of Chronic Hepatitis [27]. Specific monoclonal antibodies for IgE, CD3, CD4, CD8, CD20, and CD16 (BD Biosciences, San Jose, CA, USA) were used for immunohistochemistry [28].

Genomic DNA was extracted using a dodecyl/hexadecyltrimethylammonium bromide (DTAB/CTAB) method [29]. IL4 rs2243250, rs2070874, IL5 rs2069812, and IL13 rs20541 polymorphisms were typed by restriction fragment length polymorphism (RFLP). IL4 rs2070874 and IL13 rs20541 typing by RFLP is described elsewhere [30, 31]. The primers and restriction enzymes for IL4 rs2243250 and IL5 rs2069812 were 5′CCTAAACTTGGGAGAACATGGT, 3′TCCTCCTGGGGAAAGATAGA (AvaII) and 5′TTCCTGCTGCTCATGAACAGAATACGT, 3′CATTTTGATGGCTTCAGTGACTCTTCC (RsaI), respectively. IL4 rs2227284 and IL4RA rs1805011 polymorphisms were typed by ASPCR (allele-specific polymerase chain reaction). Primers for IL4RA rs1805011 have been described [32] and primers used for IL4 rs2227284 were 5′TTGGGTGGACAAGTAGTTGGAGCG, 5′TTGGGTGGACAAGTAGTTGGAGCT and 3′ATGTCCCATCCTGCCCAGGATAG.

### 2.1. Statistical Analysis

All statistical analyses were carried out using GraphPad Prism 5 or SPSS, v.13 Software. The clinical and laboratory parameters were analyzed using Student's *t*-test or Fisher's exact test, as well as the Mann-Whitney test where necessary. *P* values under 0.05 were considered as significant. The power was estimated for all studied SNPs and values ranged from 76 to 82%, indicating adequate sample size. In addition, all SNPs were in HWE and, as expected, Haploview analysis confirmed that the three studied IL4 SNPs were in linkage disequilibrium.

For the possible genetic associations, *χ* ^2^ or exact Fisher's test were applied. Unpaired *t*-test was used to evaluate associations between IgE and the genotypes of all studied SNPs. For regression analysis, variables presenting *P* value <0.100 in the univariate analysis were included. To identify possible gene-gene interactions, a binary logistic regression was performed considering changes in the OR.

## 3. Results

The majority of the AIH patients were classified as type 1 (85% versus 15% type 2). The median age of diagnosis was 8.2 and 4.8 years, respectively, for AIH-1 and AIH-2. In addition, 54% (13/24) of AIH-2 patients developed the disease before the age of 5 years, whereas this occurred only in 8/117 (7%) of AIH-1 patients (*P* < 0.001). Twenty-three (20%) AIH-1 and 11 (46%) AIH-2 patients (*P* = 0.006) had relatives presenting autoimmune diseases. In addition, median serum alanine aminotransferase values were higher in the AIH-2 group (28 versus 18 × upper normal limit; see Table 1).

**Figure 1:**
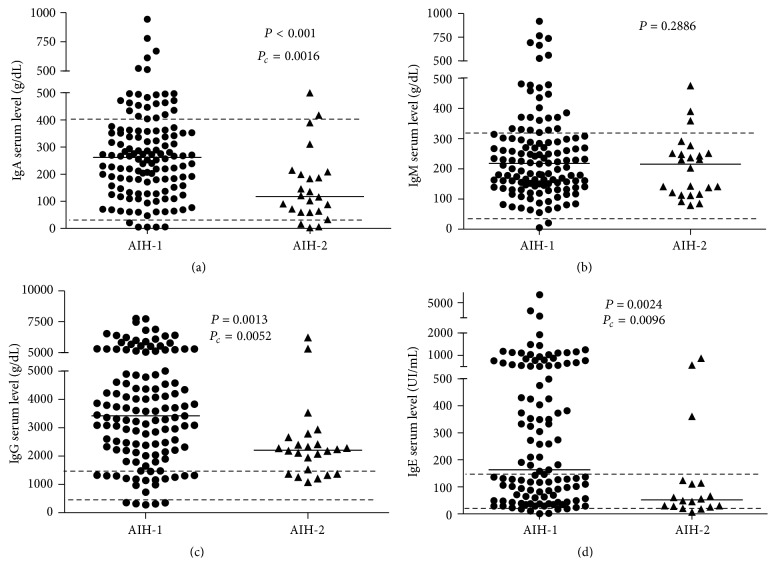
Immunoglobulins concentrations according to autoimmune hepatitis type. (a) IgA (g/dL); (b) IgM (g/dL); (c) IgG (g/dL), and (d) IgE (UI/mL). The immunoglobulins concentrations were assessed by nephelometry. Statistical analysis by Mann-Whitney nonparametric test (for medians).

Serum IgG, IgA, and IgE levels were significantly higher in AIH-1 in comparison to the AIH-2 group of patients (Figure 1). High IgE levels were observed in 50/91 (55%) of patients with AIH-1, but only in 2/17 (12%) of those with AIH-2 (*P* = 0.003) (Table 1).

Histopathology showed presence of cirrhosis in the majority of AIH-1 patients (57 out of 60) analyzed, usually accompanied by necroinflammatory activity corresponding to a score 3 and a score 4 panacinar necrosis. Liver cell rosettes were also present in almost 90% of livers, accompanied by infiltrating eosinophils and/or plasma cells, independently of patients IgE serum levels (Table 2). Importantly, in contrast to increased IgE serum levels present in about half of the patients, liver IgE was absent in only 4 of the 46 AIH-1 patients. Finally, most patients exhibited CD8^+^ cytotoxic T cell and NK infiltrating cells, in some cases without detectable CD4^+^ helper T cells (Table 3). However, irrespective of serum IgE levels, in most patients, moderate to high infiltration levels of CD4^+^ helper T cells usually accompanied by moderately elevated liver NK cells were in fact present. In conclusion and in spite of having analyzed only a subgroup (46/60) of patients, our results clearly show that the well-known infiltrating proinflammatory cell profile coexists side by side with IgE, eosinophils, and the plasma cells possibly involved in IgE production. The reason for this mixed cell profile is currently unknown.

Among the studied SNPs in AIH-1, two functionally relevant SNPs present, respectively, in the IL13 gene and in its receptor IL4RA disclosed statistically significant increases. The first SNP is IL13 rs20541 (31 versus 23% of HC; *P* = 0.024, OR = 1.55) and, moreover, homozygosis for the A allele at IL13 rs20541, known to impact upon receptor ligand affinity, was also significantly increased compared to healthy controls (*P* < 0.001, OR = 4.62). Increased frequencies were also found for A allele at IL4RA rs1805011 (68% versus 49%; *P* < 0.0001, OR = 2.15) and homozygosis for A (47% versus 19%; *P* < 0.001, OR = 3.75) (Table 4). The remaining polymorphisms did not show any relevant difference when AIH-1 and HC groups were compared (Supplementary Table 1 in Supplementary Material available online at http://dx.doi.org/10.1155/2015/679813).

Finally, we carried out analysis using a logistic regression model, which included allele carriage of the different SNPs as well as clinical and laboratory parameters. Three modes of analysis were tested. In the first mode (mode 1), presence of disease was considered as the dependent variable. The results confirmed the findings for both IL13 rs20541 (OR = 9.45 (95% 2.28–39.18) *P* = 0.002) and IL4RA rs1805011 (OR = 3.72 (95% 1.78–7.77) *P* < 0.001). To investigate a possible association of SNPs with pathogenesis of the disease, a second mode (mode 2) of analysis considered each SNP as the dependent variable. The T allele at IL5 rs2069812 showed association with treatment suspension (remission by clinical and laboratory standards) (*P* = 0.004) but was a very rare outcome, present only in 7 patients (7/117, 6%) where 5 achieved regression of fibrosis after treatment (grades IV to II). IL5 is directly involved in eosinophil activation and is a key molecule in allergy and eosinophilic inflammation [33], expressed by CD4^+^ helper T and B cells, mast cells, and eosinophils. It remains to be seen if an extended analysis confirms this indication. Finally, IgE was considered as the dependent variable in another analysis (mode 3). The presence of the T allele at IL4 rs2227284 showed association with high IgE levels (OR = 7.42 (95% CI 1.33 to 41.34), *P* = 0.02) (Table 5), an expected result.

The genes individually associated with susceptibility to the disease were examined for potential gene-gene interactions. Gene-gene interactions considered grouped genotypes for IL4RA rs1805011, IL4 rs2243250, IL4 rs2070874, rs2227284, IL13 rs20541, and IL5 rs2069812 and the presence of ^*∗*^03 and/or ^*∗*^13 alleles at the HLA-DRB1 locus. Individuals presenting homozygosis for the A allele at IL4RA rs1805011 and HLA-DRB1^*∗*^03 and/or ^*∗*^13 allele were at six times greater risk to develop the disease (OR = 14.00, *P* < 0.001) compared to the risks conferred by the same alleles individually (HLA-DRB1^*∗*^03 and/or ^*∗*^13, OR = 8.28; IL4RA rs1805011, OR = 3.72). Individuals homozygous for the A allele at IL13 rs20541, combined with HLA-DRB1^*∗*^13 and/or ^*∗*^03 allele also showed a slightly greater risk to develop the disease (OR = 8.88, *P* = 0.04).

## 4. Discussion

The recurrent presence of plasmocytes and eosinophils in liver biopsies along with the unusual finding of increased circulating IgE antibodies in Brazilian pediatric patients with AIH was the basis for this retrospective study. To further understand if those cells might be disease markers for AIH-1, we investigated gene polymorphisms of cytokines involved in plasmocyte and eosinophil maturation and IgE production. Our hypothesis was that these SNPs might play an additional role in the development of AIH, a disease primarily caused by autoreactive T cells, acting as disease modifiers in synergy with the strongly associated MHC class II HLA-DRB1^*∗*^13 and ^*∗*^03 alleles in the Brazilian admixed population [34]. Our cross-sectional analysis of laboratory and clinical parameters aimed to distinguish if the increased levels of circulating IgE are markers for the presence of an autoimmune process and therefore present in all patients irrespective of other markers or an indicator of a pathogenic role varying according to disease severity. It is also possible that IgE levels are simply an epiphenomenon caused by widespread inflammatory and immune activity.

The degree of portal inflammation and, especially, parenchymal lesions and interface necroinflammatory activity were remarkable in AIH-1 patients and occurred irrespective of IgE serum levels. A major feature in the present series of analysis was the finding of panacinar necrosis in about half of all patients, again regardless of IgE serum levels, eosinophil count, or other histology characteristics. In spite of liver-infiltrating eosinophils in about 60% of these patients, eosinophil count in peripheral blood of all patients was in the normal range (data not shown). This observation is in accordance with the observed lack of RAST reactivity in the patients. We concluded that despite the high IgE serum levels, the laboratory and clinical findings are not indicative of a concomitant allergy or atopy occurring in these children. In addition, eosinophils have a circulating half-life of only a few hours, with rapid removal of tissues by leukocyte extravasation [35]. In tissues, eosinophils live from 2 to 14 days, especially in liver and spleen. Eosinophils are not usually present in livers from healthy or CMV-infected patients, in contrast to liver transplanted patients, where eosinophil count correlates with degree of rejection [36]. In our patients, and arguably due to the widespread inflammation, not only were eosinophils present but also IgE was identified in most biopsies analyzed. In addition, plasma cells, T and B lymphocytes, and NK cells were also found in the liver of most patients, confirming the generalized inflammatory process. In AIH, T cell-mediated cytotoxicity is believed to be the central mechanism responsible for hepatic damage. In fact, the intriguingly mixed immune profile included also the clearly defined CD8^+^ cytotoxic T cell periportal infiltration responsible for the piecemeal necrosis that is a hallmark of the disease whereas CD4^+^ helper T cell and B cells gathered around portal tracts. Of note, in our group of patients, we observed a more modest score in the case of CD4^+^ helper T cell infiltrating cells than described elsewhere [24].

Our data are similar to a recent study in adult AIH and drug-induced liver injury patients. Infiltrating liver cells were profiled and the presence of eosinophils was detected after standard staining in varying percentages in both groups of patients [37], but tissue IgE was not measured. Added to the unambiguous detection of eosinophils in the biopsies of our group of pediatric patients, we show that liver IgE is present in the vast majority of patients.

Taken together, beyond the characteristic portal and periportal inflammatory cell profile, the ubiquitous presence of IgE deposits, plasma cells, and eosinophils suggests a yet unidentified additional role in the pathogenesis of AIH. In rheumatoid arthritis, the involvement of eosinophils [38] has been linked to IL-5 and TGF-*β*1, profibrogenic cytokines that contribute to collagen accumulation in tissues [39]. It is possible that, likewise, the excess liver-infiltrating eosinophils take part in the development of the severe fibrosis typical of the disease in young children.

On the other hand, IL4 and IL13 are major cytokines involved in IgE synthesis by B cells [17] and exhibit overlapping functions due to the interaction with the type II receptor composed of the IL4R*α* and IL13R*α*1 expressed in nonhematopoietic cells and shared by both cytokines [40]. IL13 additionally impacts upon tissue eosinophilia, tissue remodeling, and fibrosis, especially in the liver [17]. We observed an association between presence of the IL13 codon 110 A allele (coding for glutamine) and susceptibility to AIH-1. This variant has been associated with increased IgE levels in both atopic and healthy children [41]. Association with the functional polymorphism coding for valine in the alpha chain of the IL4 receptor was also identified (see multivariate analysis, model 1). Chen et al. (2004) [42] have previously shown that the IL13 glutamine carrying variant displays increased activity compared to the wild type arginine variant. Furthermore, they showed that signal transduction by the variant was further enhanced when the IL4 receptor alpha chain carried valine in position 50. The results suggest that the joint presence of these two polymorphisms in AIH pediatric patients may indeed impact AIH pathology and contribute to disease severity. It is possible that the presence of higher circulating and liver IgE reflects an overall stimulus of the immune system that results in enhanced immunoglobulin levels, which could include target-driven autoantibodies. It remains to be seen if any specific autoantigen is recognized by these IgE antibodies, but without a defined target this analysis remains difficult to be achieved.

The IL4 rs2243250, rs2070874, and rs2227284 SNPs included in this study have been shown to impact IL4 transcriptional activity [43] and IL4 rs2227284 (G>T), which resides in a putative transcription factor binding site, may act independently to regulate IL4 transcription and IgE production. Furthermore, presence of the T allele at IL4 rs2227284 has been associated with higher IgE levels in White, African-American, and Hispanic asthma patients [40]. In the multivariate analysis (see model 3), the same T allele was significantly associated with serum IgE levels strengthening our hypothesis of an additional role for the IL4, IL13 cytokine pathway in the pathogenesis of AIH.

## 5. Conclusion

In conclusion, in agreement with the recurrent observation of high serum IgE levels and presence of eosinophils, plasmocytes, and IgE in the liver of AIH-1 pediatric patients, we have identified novel associations with polymorphic variants of the IL13 gene and the functionally related IL4 receptor alpha chain which suggest IgE-linked immune responses may be involved in the overall susceptibility to AIH-1.

## Supplementary Material

Supplementary Table 1. Genotype and allele frequencies of IL4 (rs 2243250, 2070874, and 222702840) and IL5 (rs 2069812) polymorphisms do not show significant differences between AIH-1 and HC groups.

## Figures and Tables

**Table 1:** Clinical and laboratory findings of children with type 1 and type 2 autoimmune hepatitis.

	AIH-1	AIH-2
	*n* = 117	*n* = 24
*Clinical features*		
Age onset; median (min–max)	8.2 (1.6–15.2)	4.8 (11.1–9.0)
Sex; *n* (F/M)	78/39	21/3
Onset; *n* (acute/insidious)	98/19	20/4
Concurrent autoimmune disease^1^; *n* (%)	15 (12.8)	3 (12.5)
Autoimmune diseases in relatives^2^; *n* (%)	23 (19.6)	11 (45.8)

*Laboratory findings*		
AA: type 1, SMA/ANA/SMA + ANA; type 2, LKM (*n*)	64/7/46	24
Alanine aminotransferase IU/L (× upper normal limit); median (min-max)	18 (2–128)	28 (4–85)
Albumin g/dL; median (min-max)	3.3 (2.2–5.1)	3.5 (2.6–4.7)
Bilirubin mg/dL; median (min-max)	3.3 (0.3–27.2)	5.8 (0.6–35)
*γ*-globulin g/dL; median (min-max)	3.4 (0.9–6.3)	3.3 (0.9–4.8)
IgE IU/mL; median (min-max)	96 (11–2245)	65 (6–560)

*Histological features*		
Cirrhosis; *n* (yes/no)	64/41	10/10
Not done	12	4

F = female; M = male; AA = autoantibody; SMA = smooth muscle antibody; ANA = antinuclear antibody; LKM = Liver Kidney Microsomal;  *n* = number of individuals.

Normal albumin = 3.5–5.0 g/dL; normal bilirubin ≤ 1.1 mg/dL; normal *γ*-globulin = 0.7–1.6 g/dL; normal IgE = 20–100 IU/mL.

^1^Vitiligo, thyroiditis, diabetes mellitus, psoriasis, or Behçet's disease.

^2^First degree relatives.

**Table 2:** Semiquantitative assessment of the histopathological variables by serum IgE levels in AIH-1 and AIH-2 patients.

Histopathological variables	Score	AIH-1		AIH-2	
		IgE		IgE	
		Normal	Increased	Normal	Increased
		*n* = 27 (%)	*n* = 33 (%)	*n* = 11 (%)	*n* = 2 (%)
Structural changes	1-2	2 (7)	1 (3)	1 (9)	0 (0)
	3	2 (7)	2 (6)	1 (9)	1 (50)
	4	23 (86)	30 (91)	9 (82)	1 (50)

Portal inflammation	1-2	8 (30)	11 (33)	5 (45)	0 (0)
	3	15 (55)	11 (33)	4 (36)	1 (50)
	4	4 (15)	11 (33)	2 (18)	1 (50)

Periportal inflammation	1-2	6 (22)	7 (21)	3 (27)	0 (0)
	3	11 (41)	10 (30)	3 (27)	1 (50)
	4	10 (37)	19 (55)	5 (46)	1 (50)

Panacinar necrosis	Present	12 (44)	21 (64)	3 (27)	2 (100)

Plasmocytes	Present	20 (74)	27 (82)	8 (73)	1 (50)

Eosinophils	Present	16 (59)	19 (58)	7 (64)	1 (50)

Rosettes	Present	24 (89)	29 (88)	10 (91)	2 (100)

1 = minimal portal fibrosis; 2 = moderate portal fibrosis; 3 = bridging fibrosis; 4 = cirrhosis.

**Table 3:** Immunohistochemical analysis for tissue IgE, liver-infiltrating T and B lymphocytes, and NK cells in the liver of AIH-1 patients, grouped according to serum IgE levels.

Infiltrate	AIH-1	
	IgE serum levels	
	Normal	Increased
	*n* = 26 (%)	*n* = 20 (%)
	IgE	
Negative	3 (12)	1 (5)
Low	11 (42)	11 (55)
Moderate/elevated	8 (31)	8 (40)
Not done	4 (15)	0

	CD3	
Negative	0	0
Low	6 (23)	6 (30)
Moderate/elevated	20 (77)	14 (70)
Not done	0	0

	CD8	
Negative	0	1 (5)
Low	14 (54)	9 (45)
Moderate/elevated	10 (38)	9 (45)
Not done	2 (8)	1 (5)

	CD4	
Negative	6 (23)	6 (30)
Low	9 (35)	7 (35)
Moderate/elevated	11 (42)	6 (30)
Not done	0	1 (5)

	CD20	
Negative	1 (4)	1 (5)
Low	11 (42)	10 (50)
Moderate/elevated	14 (54)	8 (40)
Not done	0	1 (5)

	CD16	
Negative	0	0
Low	13 (50)	11 (55)
Moderate/elevated	13 (50)	5 (25)
Not done	0	4 (20)

**Table 4:** Genotype and allele frequencies of *IL13* rs20541 and *IL4RA* rs1805011 in children with type 1 autoimmune hepatitis (AIH-1) and in healthy controls (HC).

	AIH-1	HC	*P*	OR	95% CI
	*n* = 117	*n* = 160			
*IL13* rs20541	*n* (%)	*n* (%)			

Genotype					
AA	18 (15)	6 (4)			
AG	37 (32)	60 (38)	0.003		
GG	62 (53)	94 (58)			
AA versus AG+GG			<0.001	4.62	1.77–12.04
Allele					
A	73 (31)	72 (23)	0.024	1.55	1.06–2.27
G	161 (69)	248 (77)			

*IL4RA* rs1805011	*n* = 88	*n* = 212			

Genotype					
AA	41 (47)	40 (19)			
AG	37 (42)	129 (61)	<0.001		
GG	10 (11)	43 (20)			
AA versus AG+GG			<0.001	3.75	2.18–6.45
Allele					
A	119 (68)	209 (49)	<0.001	2.15	1.49–3.11
G	57 (32)	215 (51)			

*IL13* codon 110 (rs20541): A allele = Q (glutamic acid) and G allele = R (arginine); *IL4RA* codon 50 (rs1805011): A allele = I (isoleucine) and G allele = V (valine); *n* = number of individuals; OR = odds ratio; CI = confidence interval.

**Table 5:** Multivariate analysis of factors associated with AIH-1, using three different models.

	Dependent variable	*P*	OR	95% CI
	AIH-1			
Model 1				
*HLA-DRB1* ^*∗*^	Different from 03 and/or 13	<0.001	8.28	3.46–19.82
	03 and/or 13			
*IL13* rs20541	AG plus GG	0.002	9.45	2.28–39.18
	AA			
*IL4RA* rs1805011	AG plus GG	0.001	3.72	1.78–7.77
	AA			

	IL5 rs2069812			
Model 2				
Treatment suspension	Yes^a^	0.004	6.41	1.83–22.44
	No			

	IgE levels			
Model 3				
*IL4* rs2227284	TT and GT	0.022	7.42	1.33–41.34
	GG			

Dependent variable in model 1: AIH-1 susceptibility.

Dependent variable in model 2: *IL5* rs2069812.

Dependent variable in model 3: IgE levels.

*IL13* codon 110 (rs20541): A allele = Q (glutamic acid) and G allele = R (arginine); *IL4RA* codon 50 (rs1805011): A allele = I (isoleucine) and G allele = V (valine).

^a^Homozigosis for T allele.

## Abbreviations

AIH: Autoimmune hepatitis
MHC: Major Histocompatibility Complex
SNP: Single nucleotide polymorphism
TGF: Transforming growth factor
OR: Odds ratio
CI: Confidence interval
LD: Linkage disequilibrium
HWE: Hardy-Weinberg equilibrium.
