# Supplementary material for: Autoimmune Hepatitis in Brazilian Children: IgE and Genetic Polymorphisms in Associated Genes
Source: J Immunol Res. 2015 Nov 26;2015:679813. doi: 10.1155/2015/679813 (PMC4674601; doi:10.1155/2015/679813)
Supplement: Supplementary file 1 — Supplementary Table 1. Genotype and allele frequencies of IL4 (rs 2243250, 2070874, and 222702840) and IL5 (rs 2069812) polymorphisms do not show significant differences between AIH-1 and HC groups. [file 679813.f1.docx]

**Supplementary Table 1** – Frequency of *IL-4* and *IL-5* SNPs in children with Type 1 Autoimmune Hepatitis (AIH-1) compared with healthy controls (HC).

| **Polymorphism** | **AIH-1**  **n (%)** | **HC**  **n (%)** | ***P***  ***P* value** |
| --- | --- | --- | --- |
| *IL4* rs2243250 | n=115 | n=180 | 0.105 |
| Genotype |  |  |  |
| CC | 67 (58) | 91 (51) | 0.105 |
| CT | 30 (26) | 68 (38) |  |
| TT | 18 (16) | 21 (11) |  |
| Allele |  |  |  |
| C | 164 (71) | 250 (69) | 0.630 |
| T | 66 (29) | 110 (31) |  |
|  |  |  |  |
| *IL4* rs2070874 | n=116 | n=220 | 0.123 |
| Genotype |  |  |  |
| CC | 66 (60) | 113 (51) | 0.123 |
| CT | 28 (24) | 77 (35) |  |
| TT | 19 (16) | 30 (14) |  |
| Allele |  |  |  |
| C | 166 (72) | 303 (69) | 0.471 |
| T | 66 (28) | 137 (31) |  |
|  |  |  |  |
| *IL4* rs2227284 | n=79 | n=86 | 0.170 |
| Genotype |  |  |  |
| GG | 14 (17) | 15 (18) | 0.170 |
| GT | 44 (56) | 58 (67) |  |
| TT | 21 (27) | 13 (15) |  |
| Allele |  |  |  |
| G | 72 (46) | 88 (51) | 0.310 |
| T | 86 (54) | 84 (49) |  |
|  |  |  |  |
| *IL5* rs2069812 | n=97 | n=128 | 0.585 |
| Genotype |  |  |  |
| CC | 19 (20) | 20 (16) | 0.585 |
| CT | 55 (56) | 71 (55) |  |
| TT | 23 (24) | 37 (29) |  |
| Allele |  |  |  |
| C | 93 (48) | 111 (43) | 0.339 |
| T | 101 (52) | 145 (57) |  |
|  |  |  |  |
